# Recognition of cognitive dysfunction in hospitalised older patients: a flash mob study

**DOI:** 10.1186/s12877-023-04588-5

**Published:** 2024-01-16

**Authors:** Fleur C. W. Visser, Marlise E. A. van Eersel, Liesbeth Hempenius, Nicolaas A. Verwey, Caterina Band, Jessica M. van der Bol, Kris Boudestein, Suzanne C. van Dijk, Robbert Gobbens, Cornelis S. van der Hooft, Adriaan M. Kamper, Rikje Ruiter, Walther Sipers, Birgit N. A. Spoelstra, Josephine Stoffels, Dyane J. Stolwijk-Woudstra, Karlijn J. van Stralen, Astrid M. van Strien, Marjolein A. Wijngaarden, Marian Winters, Fijanne Strijkert, Barbara C. van Munster

**Affiliations:** 1 Department of Geriatric Medicine and Alzheimer Center Groningen, University of Groningen, University Medical Center Groningen, 9700 RB, Groningen, AA43 The Netherlands; 2 Geriatric Medicine, Medical Center Leeuwarden, Leeuwarden, The Netherlands; 3 Neurology and Geriatric Department, Medical Center Leeuwarden, Leeuwarden, The Netherlands; 4 Spaarne Gasthuis Hospital, Spaarne Gasthuis Academy, Hoofddorp, The Netherlands; 5 Reinier de Graaf Hospital, Geriatric Medicine, Delft, The Netherlands; 6 Department of Geriatric Medicine, Maasstad Hospital, Rotterdam, The Netherlands; 7 Department of Geriatric Medicine, Franciscus Gasthuis and Vlietland, Schiedam, The Netherlands; 8 Faculty of Health, Sports and Social Work, Inholland University of Applied Sciences, Amsterdam, The Netherlands; 9 Department of Geriatric Medicine, Tjongerschans Ziekenhuis, Heerenveen, The Netherlands; 10 Department of Internal Medicine, Isala Hospital, Zwolle, The Netherlands; 11 Department of Internal Medicine, Maasstad Hospital, Rotterdam, The Netherlands; 12 Department of Geriatric Medicine, Zuyderland Medical Center Sittard-Geleen, Heerlen-Sittard-Geleen, The Netherlands; 13 Department of Geriatric Medicine, Meander Medisch Centrum, Amersfoort, The Netherlands; 14 Department of Internal Medicine, Amsterdam UMC, Vrije Universiteit Amsterdam, Amsterdam Public Health Aging & Later Life, Amsterdam, The Netherlands; 15 Department of Geriatric Medicine, Jeroen Bosch Hospital, ‘s-Hertogenbosch, The Netherlands; 16 Leiden University Medical Center, Internal Medicine, Section Geriatrics, Leiden, The Netherlands; 17 Departments of Internal Medicine and Geriatrics, Isala Hospital, Zwolle, The Netherlands

**Keywords:** Cognitive dysfunction, Hospital admission, Older patients, Dementia, Delirium

## Abstract

**Background:**

It is important that healthcare professionals recognise cognitive dysfunction in hospitalised older patients in order to address associated care needs, such as enhanced involvement of relatives and extra cognitive and functional support. However, studies analysing medical records suggest that healthcare professionals have low awareness of cognitive dysfunction in hospitalised older patients. In this study, we investigated the prevalence of cognitive dysfunction in hospitalised older patients, the percentage of patients in which cognitive dysfunction was recognised by healthcare professionals, and which variables were associated with recognition.

**Methods:**

A multicentre, nationwide, cross-sectional observational study was conducted on a single day using a flash mob study design in thirteen university and general hospitals in the Netherlands. Cognitive function was assessed in hospitalised patients aged ≥ 65 years old, who were admitted to medical and surgical wards. A Mini-Cog score of < 3 out of 5 indicated cognitive dysfunction. The attending nurses and physicians were asked whether they suspected cognitive dysfunction in their patient. Variables associated with recognition of cognitive dysfunction were assessed using multilevel and multivariable logistic regression analyses.

**Results:**

347 of 757 enrolled patients (46%) showed cognitive dysfunction. Cognitive dysfunction was recognised by attending nurses in 137 of 323 patients (42%) and by physicians in 156 patients (48%). In 135 patients (42%), cognitive dysfunction was not recognised by either the attending nurse or physician. Recognition of cognitive dysfunction was better at a lower Mini-Cog score, with the best recognition in patients with the lowest scores. Patients with a Mini-Cog score < 3 were best recognised in the geriatric department (69% by nurses and 72% by physicians).

**Conclusion:**

Cognitive dysfunction is common in hospitalised older patients and is poorly recognised by healthcare professionals. This study highlights the need to improve recognition of cognitive dysfunction in hospitalised older patients, particularly in individuals with less apparent cognitive dysfunction. The high proportion of older patients with cognitive dysfunction suggests that it may be beneficial to provide care tailored to cognitive dysfunction for all hospitalised older patients.

## Introduction

Cognitive dysfunction is prevalent among hospitalised older patients. Prevalence rates range from 38 to 50%, depending on factors such as the age of the study population and type of hospital ward [1–4]. Cognitive dysfunction may be pre-existent, due to dementia or stroke for example [5, 6]. Also, cognitive dysfunction may be of new onset during admission, as a result of acute illness, medication (such as opioids), or the manifestation of delirium [7–9]. Older patients with cognitive dysfunction during hospitalisation are at increased risk of developing several complications, such as dehydration, falls, or infections. Additionally, patients with pre-existent cognitive dysfunction are at higher risk for developing delirium [10]. Further, the presence of cognitive dysfunction is associated with prolonged hospital stays, readmissions, institutionalisation, and mortality [4, 10–12]. Considering these risks, the presence or onset of cognitive dysfunction impacts the care needs of these patients during and after hospitalisation. Therefore, adequate recognition of cognitive dysfunction by healthcare professionals will facilitate proactive hospital care addressing these risks with a patient-centred, instead of a disease-oriented approach [4].

However, cognitive dysfunction in hospitalised patients appears to be under-recognised by healthcare professionals [3, 13]. Only two studies examined how well cognitive dysfunction is recognised by healthcare providers in hospitalised older patients. In a cohort of the university-affiliated Wishard Memorial Hospital (United States), documentation of cognitive dysfunction was lacking from the medical records of 61% of patients with cognitive dysfunction aged 65 or older [3]. In addition, recognition by physicians and nurses occurred in 44–64% of 77 patients with cognitive dysfunction aged 60 years and older who were admitted to the general internal medicine department of a university hospital in Sweden [13]. Patients in whom cognitive dysfunction was recognised were older, had fewer comorbidities, and had more severe cognitive problems [3]. Thus, to date, recognition of cognitive dysfunction in hospitalised older patients has only been investigated by studying medical records in mono-centre university hospital settings.

However, reviewing medical records may not definitively confirm whether cognitive dysfunction is truly unrecognised, as a documentation gap may be an alternative explanation. To address this issue, we evaluated healthcare professionals’ recognition of cognitive dysfunction directly at the bedside. Our aim was to investigate the prevalence of cognitive dysfunction in hospitalised older patients, and the percentage of patients in which cognitive dysfunction was recognised by healthcare professionals. Additionally, we examined variables possibly associated with recognition of cognitive dysfunction, e.g. ward type, patient characteristics, and work experience of healthcare professionals.

## Methods

### Study design and setting

A multicentre, nationwide, cross-sectional observational study was conducted on a single day using a flash mob research design [14]. The research questions and protocol (NCT: NCT05395559, 27/05/2022) were initiated by a steering committee with representatives of the University Medical Center Groningen (UMCG) and Alzheimer Center Groningen (ACG). The Medical Ethics Review Board of the University Medical Center Groningen (METc UMCG) confirmed that the Medical Research Involving Human Subjects Act did not apply (reference number 2022/083). Subsequently, the University Medical Center Groningen Medical Ethics Committee reviewed and approved the research protocol (reference number 202200087). In every participating hospital, local investigators obtained local institutional review board approval and coordinated data collection. The study was part of the ABOARD-project: A Personalized Medicine Approach for Alzheimer’s Disease [15, 16].

Data were collected on World Alzheimer’s Day 2022, the 21st of September, between 8:00 AM and 5:00 PM in three university hospitals and ten general hospitals in the Netherlands. Collaboration with a large group of data collectors (‘mob’) enabled us to collect data in a single day (‘flash’). This single-day approach ensured that the results on recognition of cognitive dysfunction would not be influenced by any learning effect or preparation on the part of healthcare professionals. Data collectors were healthcare education students (e.g., nursing, medicine, applied psychology) or hospital staff (e.g., nurses, nurse practitioners, researchers, physicians) not involved in the care of the participating patients on the day of the flash mob study. The steering committee and neuropsychologists developed standard operating procedures for data collectors and a video instruction on how to administer the cognitive test.

### Participants

Dutch-speaking patients aged ≥ 65 years who were admitted to one of the participating hospitals at the starting time of data collection were eligible for enrolment. Patients were excluded if they were unable to provide informed consent, unable to perform a cognitive test due to severe sensory impairment or severe illness, required medical isolation, or were not willing to participate. Written informed consent was obtained from all included patients. Characteristics collected from patients included age, gender, and the medical department to which they were admitted.

### Cognitive performance

The Mini-Cog was used to assess cognitive dysfunction [17, 18]. This brief screening instrument consists of a three-item word memory test and a clock drawing test. The Mini-Cog covers the cognitive domains of memory, visuospatial and executive functions. We found the Mini-Cog suitable for a flash mob study because administration requires minimal training, takes less than five minutes, and the diagnostic value was previously found not to be influenced by education level [17]. For this study, a cut-off of < 3 points out of 5 was used since scores of 0–2 indicate cognitive dysfunction based on previous findings [18]. Master students of neuropsychology with experience in neuropsychological testing scored the Mini-Cog test items independently from the test administration and calculated a total test score. In case of doubt about the score, an experienced neuropsychologist was consulted to reach a consensus score.

### Recognition

On the day of data collection, the attending physicians and nurses were asked whether they suspected the patient to have cognitive dysfunction. A yes/no answer was required. In addition, categorised years of work experience were collected from them. Data collectors ensured the Mini-Cog was administered when the nurse and physician were not present in the patient’s room. Also, the nurses and physicians did not have access to Mini-Cog results.

### Statistical analysis

Continuous parametric data are presented as mean and standard deviation (SD), and nonparametric data as median and interquartile range (IQR). Categorical variables are presented as percentages. Patients were either considered as having ‘cognitive dysfunction’ (Mini-cog score < 3) or having ‘no cognitive dysfunction’ (Mini-Cog scores ≥ 3). Differences between the two groups were tested by the independent-samples t-test for parametric data and by the Mann–Whitney U test for nonparametric data. Differences in two groups of categorical variables were tested by the Chi-Square test, and if groups were too small, by Fisher’s exact test. Differences in proportions were tested by the Z-test for proportions.

Univariable, multivariable, and multilevel logistic regression analyses were performed to identify variables associated with recognition of cognitive dysfunction by healthcare professionals. Recognition of cognitive dysfunction was the binary dependent variable, which is defined as patients with an abnormal Mini-Cog test score who are correctly recognised as having cognitive dysfunction. Independent variables were patient age, gender, Mini-Cog score, work experience of the healthcare professional, hospital type, and medical department to which the patient was admitted. For the variable ‘medical department’, we pooled together the data from wards with the same medical specialisation across different hospitals. The variables patient gender, work experience of the healthcare professional, hospital type, medical department, and Mini-Cog score were added as categorical variables. Patient age was added as a continuous variable. The multivariable regression analyses were conducted using a forward stepwise approach. A possible association between the independent variables and the dependent variable ‘recognition of cognitive dysfunction’ was separately tested for physicians and nurses. Associations were presented as odds ratios with 95% confidence intervals. A two-tailed p-value of less than 0.05 was considered statistically significant. Statistical analyses were performed using R version 4.2.1.

## Results

### Study population

**Fig. 1:**
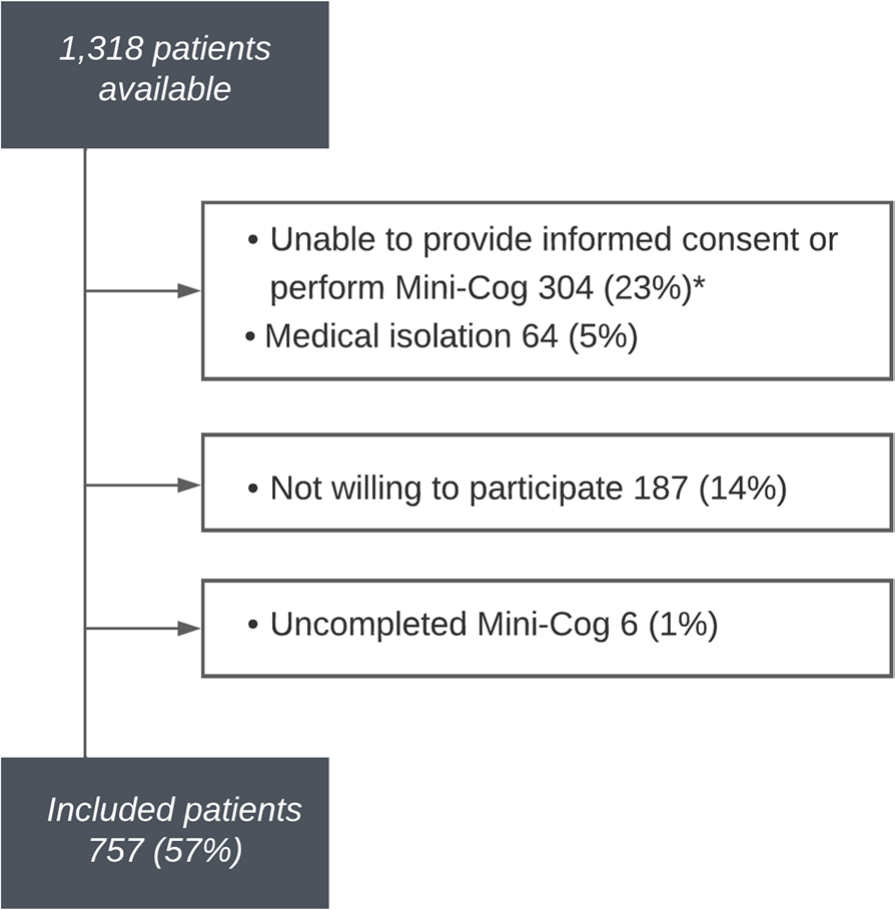
Flow chart showing patient inclusion. * Due to severe sensory impairment or severe illness

A total of 1,318 Dutch-speaking, hospitalised patients aged ≥ 65 years old were eligible for participation. Of these, 304 patients (23%) were excluded due to severe sensory impairment or severe illness, 64 (5%) because of medical isolation, and 187 (14%) because they were unwilling to participate. After consent, six patients (1%) dropped out due to uncompleted assessments. Finally, 757 patients (57%) were included (Fig. 1). The median age was 77 years (IQR 71–82), and 369 (49%) participants were female (Table 1). There were no differences between included and excluded patients with respect to age, gender, and type of ward (medical vs. surgical). Further, such differences were also not found between included patients vs. patients who were unwilling to participate (data not shown).

**Table 1:** Characteristics of the study population

	Full Cohort ***N*** = 757	Mini-Cog < 3*** N*** = 347	Mini-Cog ≥ 3 ***N*** = 410	***P*-values**
Age (years), median (IQR)	77 (71–82)	80 (74–85)	75 (70–80)	< 0.001
Female (%)	369 (49)	183 (53)	186 (46)	0.05
Hospital type, n (%)				
General	675 (89)	320 (92)	355 (87)	0.02
University	82 (11)	27 (8)	55 (13)	
Medical department*, n (%)				
Cardiology	118 (16)	55 (16)	63 (15)	< 0.001
Gastroenterology & hepatology	59 (8)	26 (7)	33 (8)	
Internal Medicine	127 (17)	56 (16)	71 (17)	
Neurology	67 (9)	50 (14)	17 (4)	
Orthopedics	58 (8)	24 (7)	34 (8)	
Pulmonary Medicine	67 (9)	21 (6)	46 (11)	
Surgery	156 (21)	62 (18)	94 (23)	
Geriatrics	44 (6)	30 (9)	14 (3)	
Mini-Cog score, n (%)				
Total score 0	103 (14)			
Total score 1	101 (13)			
Total score 2	143 (19)			
Total score 3	150 (20)			
Total score 4	109 (14)			
Total score 5	151 (20)			

*Abbreviations*: *IQR* Interquartile range. * Selection of medical departments with *n* > 15 in the Mini-Cog < 3 group

### Cognitive dysfunction

Of 757 patients in total, 347 patients (46%) scored below the cut-off value of 3 points on the Mini-Cog. Patients who scored < 3 points were significantly older compared to patients with a Mini-Cog score ≥ 3 (median [IQR], 80 [74–85] years vs. 75 [70–80] years; *p* < 0.001), and a higher percentage of these patients were female (53% vs. 46%; *p* = 0.05). Patients with a score < 3 on the Mini-Cog were more often admitted to a medical department than a surgical department compared to patients with a Mini-Cog score ≥ 3 (70% vs. 61%; *p* = 0.01). Patients with a Mini-Cog score < 3 also resided more often in a general hospital than a university hospital compared to patients with a Mini-Cog score ≥ 3 (92% vs. 87%; *p* = 0.02) (Table 1).

### Recognition by healthcare professionals

**Fig. 2:**
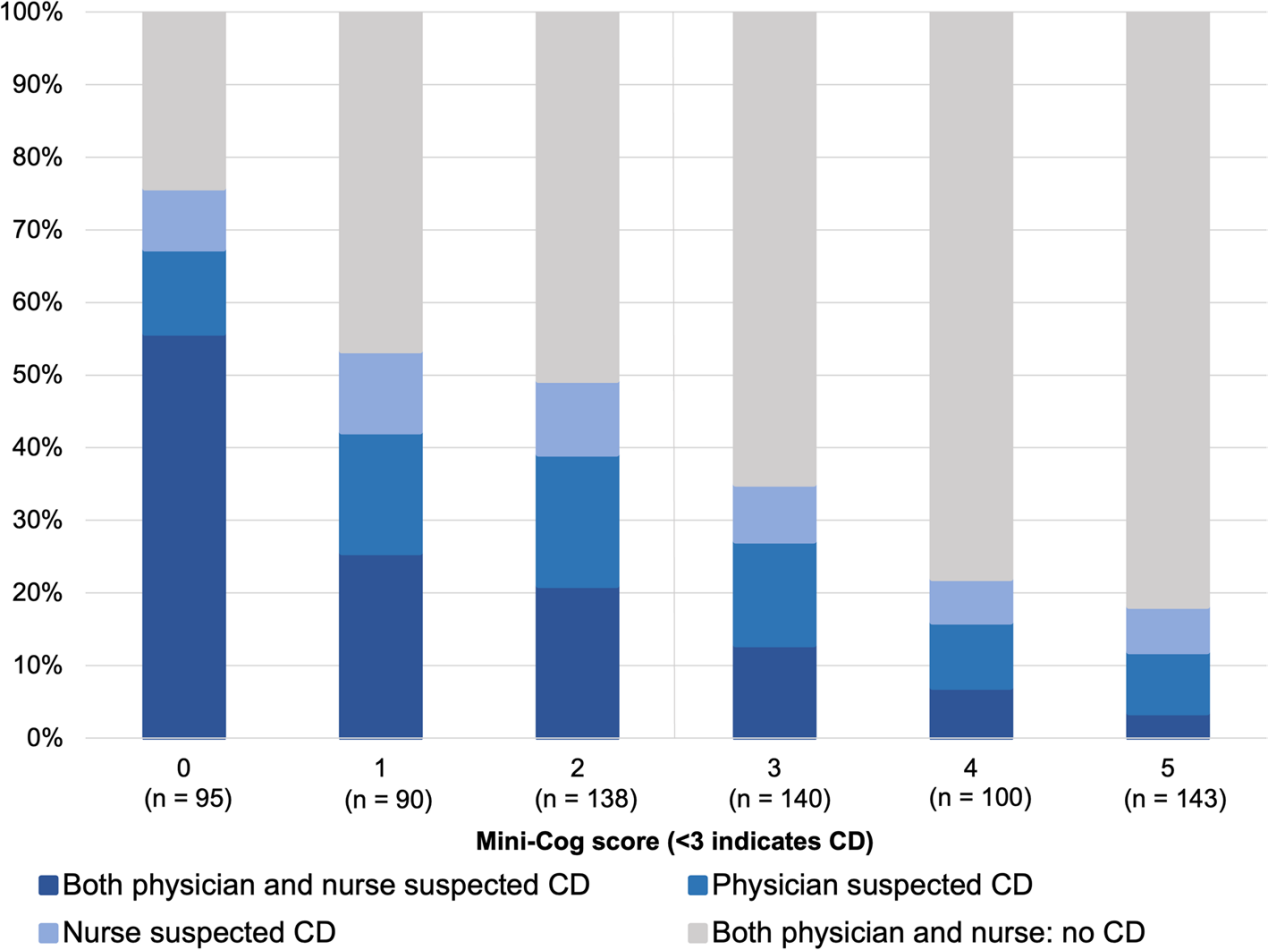
Recognition of cognitive dysfunction grouped by Mini-Cog result. Attending nurses and physicians were asked whether they suspected cognitive dysfunction (CD) in their patient. A yes/no answer was required

Data from both the attending nurse and physician were available from 323 of the 347 patients scoring < 3 on the Mini-Cog. Cognitive dysfunction was recognised by both nurse and physician in 105 of 323 patients (32%), by their physician only in 51 patients (16%), and by their nurse only in 32 patients (10%). In 135 patients (42%), cognitive dysfunction was not recognised by either their nurse or physician. Overall, physicians recognised cognitive dysfunction in 156 (48%) of their patients. Nurses recognised cognitive dysfunction in 135 (42%) patients. Cognitive dysfunction was better recognized by physicians as well as nurses at lower Mini-Cog scores (Fig. 2). There was no significant difference between physicians’ and nurses' recognition percentages. Patients with a Mini-Cog score < 3 were best identified in the geriatric department: recognition percentages in geriatric departments were 69% by nurses and 72% by physicians vs. overall recognition of 42% by nurses and 48% by physicians.

### Variables associated with recognition of cognitive dysfunction

There was no significant difference between the hospitals in recognition of cognitive dysfunction (Table 2 and 3, footnote). We found a significant association between recognition of cognitive dysfunction and the Mini-Cog score. The lower the Mini-Cog score, the stronger the association with recognition. This association was found for recognition of cognitive dysfunction by both physicians and nurses (Table 2 and 3). Among physicians, the multivariable regression analysis showed an association between recognition of cognitive dysfunction and the medical departments Gastroenterology & hepatology (OR: 0.25, 95% CI: 0.07–0.85, *P* = 0.03), Pulmonary Medicine (OR: 0.26, 95% CI: 0.07–0.93, *P* = 0.04), and Internal Medicine (OR: 0.35, 95% CI; 0.12–0.96, *P* = 0.04). Among nurses, the multivariable regression analysis showed an association between recognition of cognitive dysfunction and the medical departments Pulmonary Medicine (OR: 0.10, 95% CI: 0.02–0.49, *P* < 0.01), Internal Medicine (OR: 0.32, 95% CI: 0.11–0.91, *P* = 0.03), and Cardiology (OR: 0.31, 95% CI: 0.11–0.87, *P* = 0.03).

**Table 2:** Results binary logistic regression on recognition of cognitive dysfunction by physicians

Predictor variable	Univariable OR [95%CI]	Multivariable OR [95%CI]
Patient age (in years)	1.0 [0.98—1.04], *P* = 0.68	-
Patient gender		
Female	0.91 [0.59—1.41], *P* = 0.68	-
Male	Reference level	
Work experience physician (in years)		-
< 5	Reference level	
5–10	1.10 [0.57—2.12], *P* = 0.78	
11–15	1.37 [0.36—5.23], *P* = 0.64	
> 15	1.17 [0.55—2.48], *P* = 0.68	
Hospital type		-
General	Reference level	
University	0.91 [0.41—2.03], *P* = 0.82	
Medical department*		
Cardiology	0.27 [0.10—0.72], ***P*** < 0.01	0.36 [0.13—1.0], *P* = 0.05
Gastroenterology & hepatology	0.19 [0.06—0.62], ***P*** < 0.01	0.25 [0.07—0.85], ***P*** = 0.03
Internal Medicine	0.30 [0.11—0.81], ***P*** = 0.02	0.35 [0.12—0.96], ***P*** = 0.04
Neurology	0.60 [0.22—1.63], *P* = 0.32	0.72 [0.26—2.02], *P* = 0.53
Orthopedics	0.38 [0.12—1.19], *P* = 0.1	0.41 [0.13—1.34], *P* = 0.14
Pulmonary Medicine	0.22 [0.06—0.77], ***P*** = 0.02	0.26 [0.07—0.93], ***P*** = 0.04
Surgery	0.27 [0.10—0.72], ***P*** < 0.01	0.37 [0.13—1.02], *P* = 0.05
Geriatrics	Reference level	Reference level
Mini-Cog score		
0	Reference level	Reference level
1	0.35 [0.19—0.64], ***P*** < 0.001	0.36 [0.19—0.69], ***P*** < 0.01
2	0.31 [0.18—0.54], ***P*** < 0.001	0.34 [0.19—0.59], ***P*** < 0.001

*Abbreviations*: *OR* Odds ratio, 95%CI = 95% Confidence interval, *AIC* Akaike information criterion

Multilevel regression analysis showed no difference between hospitals: model with random intercept of hospitals AIC = 451.9; model without random intercept of hospitals AIC = 450.1

^*^ Medical departments with less than 15 included patients were excluded from the analysis. Adding this variable did not improve the multivariable regression model (AIC)

**Table 3:** Results binary logistic regression on recognition of cognitive dysfunction by nurses

Predictor variable	Univariable OR [95%CI]	Multivariable OR [95%CI]
Patient age (in years)	1.02 [0.99—1.06], *P* = 0.1	-
Patient gender		-
Female	1.24 [0.80—1.93], *P* = 0.34	
Male	Reference level	
Work experience nurse (in years)		
< 5	Reference level	Reference level
5–10	0.73 [0.37—1.41], *P* = 0.35	0.64 [0.30—1.35], *P* = 0.24
11–15	1.02 [0.38—2.76], *P* = 0.96	0.77 [0.25—2.38], *P* = 0.65
> 15	0.51 [0.28—0.93], ***P*** = 0.03	0.55 [0.28—1.08], *P* = 0.08
Hospital type		-
General	0.99 [0.44—2.24], *P* = 0.99	
University	Reference level	
Medical department*		
Cardiology	0.23 [0.09—0.61], ***P*** < 0.01	0.31 [0.11—0.87], ***P*** = 0.03
Gastroenterology & hepatology	0.27 [0.09—0.84], ***P*** = 0.02	0.31 [0.09—1.08], *P* = 0.07
Internal Medicine	0.29 [0.11—0.75], ***P*** = 0.01	0.32 [0.11—0.91], ***P*** = 0.03
Neurology	0.47 [0.18—1.23], *P* = 0.12	0.59 [0.21—1.67], *P* = 0.32
Orthopedics	0.32 [0.10—1.0], *P* = 0.05	0.34 [0.10—1.14], *P* = 0.08
Pulmonary Medicine	0.08 [0.02—0.36], ***P*** < 0.001	0.10 [0.02—0.49], ***P*** < 0.01
Surgery	0.37 [0.14—0.97], ***P*** = 0.04	0.54 [0.19—1.51], *P* = 0.24
Geriatrics	Reference level	Reference level
Mini-Cog score		
0	Reference level	Reference level
1	0.32 [0.18—0.59], ***P*** < 0.001	0.32 [0.17—0.62], ***P*** < 0.001
2	0.25 [0.15—0.44], ***P*** < 0.001	0.24 [0.14—0.44], ***P*** < 0.001

*Abbreviations*: *OR* Odds Ratio. 95%CI = 95% Confidence interval, *AIC* Akaike information criterion

Multilevel regression analysis showed no difference between hospitals: model with random intercept of hospitals AIC = 441.5; model without random intercept of hospitals AIC = 439.5

^*^ Medical departments with less than 15 included patients were excluded from the analysis

## Discussion

This is the first multicentre, nationwide observational study investigating the bedside recognition of cognitive dysfunction in hospitalised patients aged 65 years or older admitted to both medical and surgical departments. Nearly half of these hospitalised older patients showed cognitive dysfunction based on a brief screening test, and the majority of physicians and nurses were unaware of their patient’s cognitive dysfunction. Patients with more severe cognitive dysfunction were better recognised by their healthcare professionals.

Regarding the prevalence of cognitive dysfunction, we here confirm prior research demonstrating that a considerable proportion of hospitalised older patients shows cognitive dysfunction [1–4]. These prevalence rates may vary between studies depending on factors such as age variations across cohorts, the type of hospitals and wards studied, and the measurement methods employed.

We found lower recognition rates than previous studies, which may be attributed to variations in instruments used for measuring cognition and approaches used for measuring recognition [3, 13]. For instance, Boustani et al. retrospectively assessed cognitive dysfunction using the Short Portable Mental Status Questionnaire (SPMSQ) [19]. Their recognition rates were based on International Classification of Disease (ICD) codes registered in medical records. A cognitive dysfunction was considered recognised in patients with a medical record ICD-code indicative of cognitive dysfunction (reported at hospital admission, discharge or during the year prior to hospitalisation) as well as an abnormal SPMSQ-score. Of 424 patients with an abnormal SPMSQ-score, 61% were recognised. The recognition rates of our study, however, are based on direct inquiry of healthcare professionals at the bedside. Therefore, our findings more closely correspond to how healthcare professionals approach the patient during admission, and whether they consider cognitive dysfunction when providing care.

Surprisingly, our study found that nurses recognized cognitive dysfunction in a lower percentage of patients (42%) compared to nurses in the study of Torisson et al. (64%), while the physician recognition percentage in our study was similar (48% vs. 44%) [13]. Cognitive dysfunction was measured using the Mini-Mental State Examination (MMSE) and clock drawing test, and recognition was determined by the presence of cognitive dysfunction symptoms in the admission records of nurses and physicians. The inconsistency of the nurse recognition percentage may be due to differences in nurse education between nations [20].

Overall, the recognition rates of cognitive dysfunction by healthcare professionals are low. Our findings confirm that patients with lower cognitive test scores are better recognised as having cognitive dysfunction than those with slightly higher scores [3]. Nevertheless, patients with less noticeable cognitive dysfunction also require attention in care due to their increased risk of complications and potential issues, such as medication adherence [4, 21]. Thus, efforts to improve recognition of cognitive dysfunction during hospitalisation are necessary.

Previous research has probed several strategies for improving recognition of cognitive dysfunction, such as the use of screening instruments, observation scales, education of hospital staff, or a combination of these in hospital-wide programs [22–30]. Firstly, it can be considered to implement structural cognitive screening during hospitalisation [22]. However, to our knowledge, no previous studies demonstrated that screening instruments improve recognition of cognitive dysfunction, and thereby, the quality of hospital care. Rice et al. found that only 34.5% of included patients were screened for cognitive dysfunction, while among those unscreened, 72% were later identified as having cognitive dysfunction [31]. Healthcare professionals in the study cited lack of time as major barrier to conducting screenings. Moreover, one concern is whether standardised cognitive screenings will raise awareness among healthcare professionals. Without clear agreements on interventions for abnormal test results, screening may become a mere box-ticking exercise [32, 33]. Secondly, instead of screening instruments, observation scales may help healthcare professionals to recognise cognitive dysfunction [23, 24]. However, studies do not provide information on whether the observation scales actually improve recognition rates. Thirdly, educational intervention studies revealed some evidence of a slight increase in recognition of delirium after training healthcare professionals [25]. However, the most promising strategies are likely hospital-wide programs that combine screening, a bedside alert for cognitive dysfunction, and education in awareness and support [26–30].

Indeed, such hospital-wide intervention programs were shown to reduce hospital-acquired complications and enhance the quality of life of patients, satisfaction of their relatives, and confidence of healthcare professionals in caring for them [26–30]. One such intervention is the Dementia Care in Hospitals Program (DCHP) across four hospitals in Australia [26]. The DCHP resulted in a 14% reduction in hospital-acquired complications among the screened 65 + population, such as urinary tract infections, pressure areas, pneumonia, and delirium. This program not only improved patient care, but also the confidence of healthcare professionals in caring for patients with cognitive dysfunction. Additionally, the program led to greater satisfaction among relatives regarding the provided care [27, 28]. Also, if cognitive dysfunction is recognised, relatives can be more intensively involved in the medical treatment during the admission, which can improve hospital outcomes [34]. Involving relatives in the care process may even reduce the number of readmissions [35].

If healthcare professionals recognise cognitive dysfunction in patients, it has clinical implications when determining follow-up care after hospitalisation. Being unaware of cognitive dysfunction may lead to inadequate transition of care after discharge, resulting in negative consequences such as medication errors, reduced medication compliance, or unplanned readmissions [21, 36]. Not every patient may need follow-up care, as some patients who experience cognitive dysfunction during their hospital stay might recover once they return home. Nevertheless, it is important to distinguish between patients who show improvement in cognitive function and those who do not. Phelps et al. [33] evaluated what happened with screened patients after discharge. The patients with previously unknown cognitive dysfunction were identified as at risk for dementia through cognitive screening. In 92% of discharge letters documenting patient risk, healthcare professionals in primary care reassessed or referred the patient within six months after hospitalisation [33]. Although, generally, a well-considered follow-up plan is standard practice in recognised delirium and dementia, our results emphasise the importance of recognising cognitive dysfunction to provide adequate follow-up care for previously unknown or less obvious cases of cognitive dysfunction.

There are several strategies to improve hospital care for older patients with cognitive dysfunction. As discussed above, one possible approach is to implement interventions that enhance recognition of cognitive dysfunction and ensure that these patients receive appropriate care. Future research into the characteristics of patients with unrecognised cognitive dysfunction and underlying causes can provide valuable information in this regard. Additionally, based on our findings that nearly half of hospitalised older patients show cognitive dysfunction, it may be beneficial to provide care that is tailored to cognitive dysfunction for all hospitalised older patients.

Some limitations of this study must be noted. First, an acute illness may reduce a patient’s ability to concentrate, possibly leading to lower test scores. This may overestimate the prevalence of cognitive dysfunction. Nevertheless, even though it may be caused by reduced concentration, the lower test score is still relevant to take into account when providing care. Healthcare professionals should be aware of the patient’s functioning when discussing treatment because it affects the patient’s ability to retain information. Second, the use of a single yes/no-question may have contributed to some of the false-positive assessments by physicians and nurses, who incorrectly assigned patients as having cognitive dysfunction. Some physicians and nurses may not have been familiar enough with their patients to answer this question. Further, the question does not allow for consideration of the degree of dysfunction or other thoughts on the situation. Additionally, some modesty in drawing conclusions is appropriate since a brief cognitive screening instrument was used to compare with the judgments of nurses and physicians. The brief cognitive screening instrument was deliberately chosen because it can be easily integrated into current clinical practice, unlike extensive cognitive tests. However, brief tests inherently have limitations, such as not accounting for the influence of age and the inability to assess various cognitive domains or the aetiology. Still, the purpose of the brief cognitive test in this study was to identify patients with a high probability of cognitive dysfunction rather than a definite diagnosis. Moreover, this study used the relatively strict and validated cut-off of < 3 to reduce the risk of false-positive results [18]. This relatively strict cut-off made it unlikely that the recognition by healthcare professionals was underestimated. Third, the number of exclusions in our study can be considered as a limitation. However, this is as would be expected in an older, vulnerable study population [37]. Moreover, we found no significant differences in age, gender, and medical discipline type between the included and excluded patients. Lastly, subgroups of medical disciplines are small limiting conclusions about associations between medical disciplines and recognition of cognitive dysfunction.

Despite the limitations, our study has several important strengths. Firstly, we prospectively examined the percentage of patients in which cognitive dysfunction was recognised using direct enquiry of healthcare professionals. Therefore, our study is likely a more reliable representation of the clinical situation than medical record studies. Secondly, our study population is multicentre, nationwide, and heterogeneous, which contributes to the representativeness and generalisability of the study. Thirdly, by using a single-day flash mob design, healthcare professionals were asked on one occasion only. This eliminated potential biases from healthcare professionals' learning or preparation.

## Conclusion

To conclude, cognitive dysfunction, as measured with a brief cognitive screening instrument, is common in hospitalised older patients and is poorly recognised by healthcare professionals, particularly in individuals with less apparent cognitive dysfunction. The high proportion of older patients with cognitive dysfunction suggests that it may be beneficial to provide care tailored to cognitive dysfunction for all hospitalised older patients.

## Data Availability

The datasets used and/or analysed during the current study are available from the corresponding author on reasonable request.

## Abbreviations

SD: Standard deviation
IQR: Interquartile range
SPMSQ: Short portable mental status questionnaire
ICD: International classification of disease
MMSE: Mini-mental state examination
DCHP: Dementia care in hospitals program
